# Control of antimicrobial resistance in Iran: the role of international factors

**DOI:** 10.1186/s12889-020-09006-8

**Published:** 2020-06-05

**Authors:** Mojtaba Mehtarpour, Amirhossein Takian, Babak Eshrati, Ebrahim Jaafaripooyan

**Affiliations:** 1 Department of Health Management and Economics, School of Public Health, Tehran University of Medical Sciences, Tehran, Iran; 2 Department of Global Health and Public Policy, School of Public Health, Tehran University of Medical Sciences, Tehran, Iran; 3 Health Equity Research Centre (HERC), Tehran University of Medical Sciences, Tehran, Iran; 4 Center For Preventive Medicine, Department of Social Medicine, Iran University of Medical Sciences, Tehran, Iran

**Keywords:** AMR containment, Global health, International cooperation, Health policy, Iran

## Abstract

**Background:**

Antimicrobial resistance (AMR) is currently causing various challenges for all countries around the world. Accordingly, the WHO is placing a great emphasis on the global partnership and allinaces to drive countries towards developing policy guidances and a strategic framework for AMR contatiment. This study thus seeks to elaborate on the international factors underlying AMR management in Iran.

**Methods:**

Semi-structured interviews were conducted with managers from the Ministry of health (n = 14), Iran veterinary organization (n = 4), the national professional associations (n = 3) and researchers (n = 3), between November 2018 and July 2019. Participants were selected using purposeful and snowball sampling. Interviews were recorded and transcribed verbatim and were subsequently coded and analyzed thematically using MAXQDA software (V.18) and reported.

**Results:**

International enabling and predisposing factors were identified in relation to the AMR control in the country. Enabling factors included knowledge transfer, facilitation in policy agenda setting, formulation and implementaion process, and AMR monitoring. Predisposing factors, alternatively, encompassed the migration of infectious patients, trafficking of medicine and livestock from neighboring countries, and the imposed sanctions.

**Conclusion:**

Nowadays, AMR is taken cognizance of as a global challenge, thus to be addressed effectively, needs an international consensus more than ever. This harmony would not certainly underrate national efforts, but instead, is needed to reinforce such efforts through e.g. technical and financial assistance. It is suggested for policymakers to use all available political and legal means such as health diplomacy to establish humanitarian channels in order to enhance global convention and remove possible barriers as the sanctions and reduce their adverse consequences for AMR control.

## Background

“Antimicrobial Resistance is a crisis that must be managed with the utmost urgency. As the world enters the ambitious new era of sustainable development, we cannot allow hard-won gains for health to be eroded by the failure of our mainstay medicines.” Dr. Margaret Chan, former Director-General of the World Health Organization (WHO) said in the introduction of Global Action Plan (GAP (on AMR [1]. Moreover, she shed light on the importance of preserving antimicrobials to protect our future. Earlier in 2011, the WHO dedicated the World Health Day of AMR to warn of the danger of returning back to the pre-antibiotic era, and invited countries to take urgent actions [2].

It is forecasted that AMR will account for 10 million deaths per year globally. Low- and middle-income countries (LMICs) will be heavily affected by AMR in a direct or indirect way [3]. In 2015, the WHO nominated the Eastern Mediterranean Region as one of the weakest in combatting AMR due to the lack of national action plans, poor awareness, fragmented information systems, irregular monitoring and surveillance, weak laboratory capacity, lack of thorough national programs for infection control and patient safety, inappropriate prescribing, and counterfeit drugs and medicines [4].

Iran is one of the countries with a high rate of antimicrobial consumption [5]. This could stem from the lack of national guidelines for prescription, and uncontrolled, over-the-counter sale of medicines including antimicrobials [6]. Overuse and overprescribing of antimicrobials is a long-standing problem in Iran’s health system. Half a century ago, researchers warned of antimicrobials overuse and the resultant AMR as a future challenge for Iran’s health system [7]. This concern is still present, as the National Committee for Rational Use of Drugs’ (NCRUD (reported that more than 50% of patients have received antibiotics in 2015 [5]. Although there is no reports on the inapproriate use of antibiotics in given year, the NCRUD has considered the percentage itself irrational behavior [5]. Accordingly, Iran has reported high resistance rates in the bacteria of international concern such as *Escherichia coli* (resistance to third-generation cephalosporins (41%), or Fluoroquinolones (54%)), *Klebsiella pneumoniae* (resistance to third-generation cephalosporins (48%), or Carbapenems (54%)) [8].

Although the eradication of AMR is impossible, effective planning and management might reduce its risks and negative consequences [9]. Iran has formulated a national action plan to combat AMR which is yet to be ratified. Various organizations and institutions, Ministry of Health and Medical Education (MOHME) and Iran Veterinary Organization (IVO), both at macro and micro levels, are somehow involved in AMR surveillance and control. The MOHME has launched some initiatives including; a national nosocomial infection surveillance program, a national committee for deciding on rational prescribing and drugs usage, a national program for surveilling various pathogens, and setting antimicrobial restrictions in public and private sectors. The IVO has also started programs including antibiotics-free chicken and eggs, monitoring the drugs residues in animal source food products. Nevertheless, the growing trend of AMR in the country in previous years has encountred the health system with a major chanllenge [5, 8]. Many international guidelines and recommendations have been released with regards to AMR containment [1–3, 8, 10–12], reflecting the importance of the issue for interconnected world in which humans, animals, water and food work as transporters of resistant microorganisms. This in turn uncovers the need for an international and multisectoral coalition utilizing effective strategies such as health diplomacy for AMR management [13, 14].

The increased globalization has also borne on the national policy-making efforts (e.g. for eradication of polio, tuberculosis or HIV control) [15], paving the way for a new global dialogue, especially among the LMIC. Thus, national health policy making and analysis should take into account such complexities and also the effect of related stakeholders, as the international processes and actors could shake the national policies [14, 15]. Countries might not be able to control all ill-health related determinants without collaborating with others, therefore and in terms of globalization, there is a need to shape coordination, cooperation and collaboration on a broad-spectrum of issues to assist in developing their capacity [15].

Iran is currently involved in various international diplomatic and business relations with neibouring and other countries and also affected by such factors as sanctions by some countries [16–18]. Entrance of a large group of Afghan and Iraqi immigrants to Iran, due to their previous turbulent situations (caused by persisting wars) in previous decades, and illegal drug and human trafficking through the eastern borders with Pakistan and Afghanistan have influenced the health system in Iran [15, 19, 20].

While the Global Action Plan (GAP) emphasizes mostly on One Health approach and an international collaboration and partnership to tackle the threat of AMR [1], to the best of our knowledge, no previous research has been carried out to look into the link between international factors and AMR management in Iran. This study thus seeks to explore the enabling and predisposing effects of international factors on the AMR containment policies in Iran.

## Methods

### Study design and participants

A qualitative research was used to collect data from a wide range of stakeholders from different organizations, institutions, national societies, and associations, both at macro and micro levels, including policymakers, managers, and academics and besides the related national and local documents.

### Sampling and data collection

Participants were recruited using both purposeful and snowball sampling (n = 24). Maximum variation was considered to generate a well-rounded view on the topic. Initially, semi-structured face-to-face interviews were held, using an interview guide, with key informants. A number of 14 types of documents on AMR were also obtained and analysed from the websites of the related ministries and organisations. The interviewees, identified during the documents analysis, were invited via email and phone call. At the end of each interview, conducted by MM, participants were asked to introduce those knowledgeable in their field. The researchers refined the topic guide, after the first five interviews as pilot interviews. All interviews were undertaken in participants’ offices between November 2018 and July 2019 and lasted for about 55 min. A file containing the introduction, ethical considerations and study objectives was sent to participants, together with assurances about confidentiality and anonymity. All interviews were carried out and analysed in Persian language, digitally recorded and transcribed verbatim, except one in which notes were taken instead, because of the participant’s reluctance. Data collection continued until saturation; that is, when no new findings emerged.

### Data management and analysis

Data were analyzed based on thematic analysis [21] using MAXQDA software (version 18) to facilitate categorization, storage, and retrieval of information. While proceeding with the interviews, the analysis was already in action via reading and re-reading the transcripts and familiarization. The interview transcripts were read several times for familiarity by MM and EJP. Initial codes started to emerge shaping the initial thematic framework which was then applied to the rest of data. The codes were then categorized as two main themes and seven sub-themes, with the final classification was approved by all authors after discussing all discrepancies.

To enhance the trustworthiness, triangulation was conducted by cross-checking the themes with the relevant formal documents. Moreover, the initial findings were also sent back to some key interviewees for assuring content accuracy and clarity (member checking).

## Results

In total, 24 experts including 13 men and 11 women were interviewed (Table 1). Several international facilitators and barriers were identified as influencing AMR and its management policies in Iran. These factors have been categorized under two main themes and seven sub-theme as illustrated in Table 2.

**Table 1:** Participants’ information (p1 - p24)

Participants	No.	Characteristics
Health Deputy of MOHME	5	Policymakers at national level [1–4], middle-level managers [5]
Deputy of Curative Affairs	3	Policymakers at national level [6–8]
Food and Drug Organization	3	Middle-level organizational agents [9–11]
Health Reference Laboratory (HRL)	2	High-level managers [12, 13]
Supreme Council for Health and Food Security	1	Middle-level agents of the Ministry of Health [14]
Iran Veterinary Organization	4	Policymakers at national level [15–18]
AMR-interested researchers	3	Academics associated with research [19–21]
National Professional Associations (Iranian Society of Infection Disease and Tropical Medicine (ISIDTM), Iranian Mycological Society, Iranian Society of Microbiology)	3	Managers [22–24]

**Table 2:** Role of international factors on AMR management in IRAN

Theme	Sub-themes	Codes
**Enabling factors in the AMR containment**	Facilitating policy making process	▪ International Advocacy for the AMR agenda setting ▪ Cooperation in the establishment of national AMR surveillance system ▪ WHO Support for AMR monitoring programs ▪ The role of the Codex Alimentarius Committee (CAC) in developing a platform for promoting cross-sectoral cooperation and improving national standards
	Knowledge transfer	▪ Holding workshops and meetings ▪ Easing students’ dispatch for continuing their education
**Predisposing factors for AMR**	Neighboring Countries related issues	▪ AMR transmission risk ▪ Medicines and livestock trafficking
	Sanctions and the supply of AMR medical products and technologies	▪ Inflation of medical supplies ▪ Difficulty of payments to the foreign sellers and inward shipping
	Sanctions and stewardship	▪ Corruption and lack of transparency ▪ Increased cost of food-producing animals and the effect on antimicrobials overuse Expediency; preference for the access over quality
	Sanctions and financing	▪ Budget reduction for AMR policies ▪ More attention to the curative medicine
	Sanctions and research and knowledge sharing	▪ Quantity and quality of research on AMR ▪ Researchers’ communication with global scientific community

### Enabling factors in the AMR containment

#### Facilitating policy making process

##### International advocacy for the AMR agenda setting

The WHO, Organisation for Animal Health (OIE) and the Food and Agriculture Organization (FAO) drew the attention of health policymakers to the AMR challenge. The WHO and OIE had selected AMR and food safety as key themes for world health days in 2011, 2015 and 2017, and urged countries to adopt the recommended policies in order to control this issue. In 2015, the WHO launched the global action plan, and called on countries to develop national action plans based on their requirements and resources.

Iran, in line with the WHO, has formulated policies and developed a national action plan based on the GAP. A manager from the Deputy of Health stated *“Before we detect this issue based on research and try to address it as an important problem in the country, the WHO was the pioneer and urged countries to join and share … .. We are indebted to the WHO which drew our attention to this issue” (P5).* Another manager also mentioned *“Since 2012, the slogan of the WHO was AMR, we prioritized the issue in the country; there was a lot of coordination and cooperation; many meetings were held at the Communicable Disease Center for officials in collaboration with the* Iran Food and Drug Administration *(IFDA*)*, the Veterinary Organization and the Ministry of Agriculture, the Health Reference Laboratory, academicians and institutions experts. We aimed to enable these experts and managers to tackle the problem” (P4).* Similarly, a manager in IVO said: *“The OIE is warning about AMR crisis as it brought us to the fact of essential confrontation. The OIE’s emphasis and also the experience of other countries have alerted us.” (P18).*

##### Cooperation in the establishment of national AMR surveillance system

The WHO has played an effective role in initiating the national AMR surveillance system in Iran. According to the records of both the WHO and the MOHME, a team consisting of two experts from the WHO Office in Tehran, a leading team from WHO/EMRO Public Health Laboratories, an AMR technical team (WHO/HQ), and two experts from the Swedish Public Health Agency attended in Iran in February 2016. This consulting team and a group of professionals and managers from the MOHME visited the Health Reference Laboratory (HRL) (in the MOHME), the Iranian Center for Communicable Disease Control (CDC), Central and References and Applied Studies (in the IVO) and hospitals, as the AMR surveillance sites in Mashhad, Tehran, and Isfahan. They explored and presented the existing challenges, weaknesses, strengths and capabilities, and introduced a roadmap for the initial launch of the national AMR surveillance system and its gradual development. The CDC in collaboration with the HRL joined the program and took important steps for establishing the Iranian surveillance system using the Global AMR surveillance system (GLASS) framework. One participant from the Project Executive Team stated *“Developing a national plan in our country is a hard work. By designing a simple program, the WHO called for countries to start with. Therefore, we now have a national plan based on GLASS. Moreover, the WHO has highlighted some specific microorganisms for follow-up, but we had modified monitoring procedure of microorganisms and antimicrobials based on our circumstances, risks and available resoures” (P12).*

##### WHO support for AMR monitoring programs

Currently, the WHO has implemented the Extended-Spectrum β-Lactamase (ESBL) Monitoring Program in Iran in collaboration with the AMR Research Center at Iran University of Medical Sciences, which can generate good information on this microorganism in humans, animals and environmental ecosystems, as well as it will promote cross-sectoral coordination in implementing the national programs. One of the project’s executives declared *“At GLASS, nowadays we only collect information at the human level but this project is tricyclic as it covers animals, humans and environment. In a way, we are practicing cross-sectoral coordination and starting to implement large-scale national programs. The WHO finances the plan, supports data collection and determines the required materials, meanwhile, we provide the needed manpower. In addition, we will estimate the budget that will be allocated for implementing the aforementioned plan” (P4).*

##### The role of the CAC in developing a platform for promoting cross-sectoral cooperation and improving national standards

The CAC commission has created a good platform for cross-sectoral cooperation and improvement of national standards. Iranian National Standards Organization is a member of the CAC and starting with the Codex Intergovernmental Task Force on AMR, Iran has also taken steps to fight against AMR by localizing Codex principal texts like the code of practice to minimize and contain AMR and also developing guidelines for risk analysis of foodborne antimicrobial resistance. A member of the committee said *“implementation of national standards written by the Standard Organization depends on the link to strong executive bodies such as ministries. Now in Health Reference Laboratory, I am in charge of one of the National Mirror Codex Committees which deals with laboratories. The Codex helps to implement local standards, recommendations, and guidelines by creating a link with the Ministries of Health and Agriculture and their subordinates such as the the Reference Laboratory and the IFDA or IVO. This partnership has never been seen before or it has been in a weak status so far. For example, we didn’t work with the codex but we joined the committee. In my opinion, the way forward is better than in the past. We all have to share our experiences” (P12)*.

#### Knowledge transfer

##### Holding workshops and meetings

Strengthening the knowledge and evidence-based practice of policymakers and technical experts was the result of the cooperation of international and local organizations. The WHO Advisory Group on the Integrated Surveillance of AMR (AGISAR) members in Iran have consulted with authorities about the available infrastructure and resources to better control this issue in food chains. In this regard, a workshop was organized by the WHO in collaboration with the Research Institute for Gastroenterology and Liver Disease at Shahid Beheshti University of Medical Sciences, Food and Drug Control Reference Laboratories Center, HRL and CDC in 2015.

Surveillance and control studies of water and foodborne diseases, and increasing resistance and contributing factors to related outbreaks were also followed by the WHO instructors and Iranian experts. WHO-NET training software was provided to collect and analyze information about AMR. The workshop was attended by health deputies of universities, epidemiologists across the country, and officials at the CDC, veterinarians, environmental health professionals, and laboratory specialists. One of this workshop organizers mentioned *“These workshops were very effective in enhancing our knowledge, we have been working for many years on AMR professional tests with the WHO’s Global Foodborne Network office. We showed our expertise, interest and infrastructure, then they desired to consider us as a hub in the region in order to organize these workshops with great support of the WHO and cooperation of the MOHME” (P19).*

To comply with the reference standards, in terms of training and capacity-building programs, a workshop on new methods of screening antibiotics in food-producing animals had been held in 2018 by experts from the Rikilt Wageningen UR in Netherland in cooperation with Shahid Beheshti University of Medical Sciences in Tehran, Iran.

One of the workshop organizers stated *“Despite sanctions, memorandum of understanding has been signed with universities such as the institute for food safety RIKILT for training our laboratories technichians. In addition, they sought to maintain a relationship with us regarding food health …*. *These courses have raised the level of our expert’s knowledge” (P13).*

##### Easing students’ dispatch for continuing their education

In collaboration with universities and international institutions, many Iranian students have been enrolled in postgraduate studies in microbiology, food hygiene, veterinary medicine and biotechnology. Those graduated students could be employed in the field of AMR as lecturers, researchers and practitioners.

### The predisposing factors for the AMR

#### Bordering countries

##### AMR transmission risk

Extended borders between Iran and its neighbors Pakistan, Afghanistan, Republic of Azerbaijan, and Iraq has raised risk of AMR transmission. For instance, Iran has an old relationships with Azerbaijan and Iraq due to the cultural and religious proximity; therefore, a large number of people cross the border daily for receiving health care or getting their own needs. One interviewee from the Deputy of Health mentioned *“We have a neighboring country with more than 2 million malaria cases, more than 90% of them have been clinically diagnosed without laboratory examination … no basic treatment … they take medicines of low quality just for two or three days. So, resistance towards anti-malarials is highy expected in such country. Incidence of most malaria cases in Iran come from illegal neighboring immigrants. Also for tuberclusis, 13 percent of new cases and 17 percent of multi-drug resistant cases are due to those immigrants” (P8).*

##### Medicines and livestock trafficking

Livestock, medicines, and medical equipment trafficking from neighboring countries accelerates the AMR aggravation in Iran. A manager in the IVO stated “*Trafficking of livestock from eastern borders is a real problem and should be highlighted as an urgent. These livestock are not vaccinated and deemed as a source for bio-threats that can transmit diseases to other livestocks inside our country … …*. *Unfortunately, the way of smuggling across the borders is out of control. Now if you want any veterinary medicine, you can go to one of the shops in [……*] *square, introduce yourself as a farmer; and ask for chloramphenicol …*. *You will get it easily and in any quantity” (P16).*

#### Sanctions and the supply of AMR medical products and technologies

##### Inflation of medical supplies

The sanctions-induced inflation has also reduced the ability to provide quality medical products needed to combat AMR. An interviewee said *“During our meetings with the Minister last year, we agreed on the supplies we need. Our budget was approved when the exchange rate was 1 United States Dollar (USD) = 40000 Iranian Rial (IRR). After escalating of sanctions, IRR has dropped to one-fourth of its previous value. Now we can hardly purchase one-fourth of our needs” (P1).* In the same regard, one of the managers at the Mycological Society pointed out *“The resources constraints have grown and we all feel it …* many essential *medications are unavailable for many infections, so colleagues have been obliged to prescribe alternative medicines of low quality and more side effects. For example, we have a patient suspectus of having* aspergillosis *infection, voriconazole is the first and best choice, this drug was already expensive before sanctions and nowadays it became more expensive and hardly to be found” (P22).*

##### Difficulty of payments to the foreign sellers and inward shipping

Sanctions have led to the disruption of purchasing and importing laboratory equipment, diagnostic tools, antimicrobial drugs and vaccines in the human and animal sectors which in turn affect the central bank and money transfers, insurance, ports and shipping, and even trading movement between Iran and European and American companies. It should be noted here that some countries may seek to avoid ngative consequences resulting from any transaction with Iran such as imposing partial sanctions or reducing finaincial aids. One participant from the Deputy of Health stated *“According to the Sustainable Development Goals (SDGs), 90% of deaths, and 80% of TB incidence rates should be reduced by 2030. Such ambitious goals cannot be met in the light of current circumstances. The WHO emphasizes the access to new diagnostic tools and short-term treatments, e.g. the automated* GeneXpert *testing* device*, which characterized by high speed of microorganism detection and resistance, and less risk of contamination but the only manufacturer is American company. We barely import several devices with the United Nations (UN) assistance over many years. After the end of the global fund project, the cartridges stocked out and we hardly bought again. Unfortunately, the sanctions were escalated this year after the US Company agreed to sell us cartridges and devices but the embargo on shipping and insurance companies prevented others to ship cargo to Iran” (P1).*

#### Sanctions and stewardship

##### Corruption and lack of transparency

The increased trafficking in human and animal medicines, the import and manufacturing of low-quality, counterfeit equipment and raw materials, as the signs of corruption and low transparency, all were somehow encouraged following the sanctions, exacerbating the AMR in the country. One respondent from the IVO said *“The increased trafficking in human and animal medicines, the import and manufacturing of low-quality, counterfeit equipment and raw materials, as the signs of corruption and low transparency, all were somehow encouraged following the sanctions, exacerbating the AMR in the country” (P17).*

##### Increased cost of food-producing animals and the effect on antimicrobials overuse

The sanctions have led to the overuse of antimicrobials in the veterinary sector which resulted in the increasing AMR. Rise in the prices during the boycott conditions and government’s attempts in the meantime to preserve the meat prices at an affordable level for the consumers has coerced the farmers to increase their profit by any means; e.g. feeding antibiotics to animals to promote specifically their growth. One participant from the IVO mentioned *“With sanctions, medicines, food and transportation became expensive and accompanied with an increase in the animals’ prices. Prices announced by the government were no longer economical for the farmers, so they have to fend for themselves (the interviewee used a Persian proverb (by cheating. They use more antibiotics to avoid casualties, and especially cheap ones to reduce the expenses … the farmers think that they can’t raise the price but try to promote the weight gain by adding antibiotics to animals food” (P17).*

##### Expediency; preference for the access over quality

Sanctions has hampered importing medicines and vaccines, equipment, and animal feeds. This has somewhat led to a rise in the market prices. In such conditions, accessibility could become more important to the government than food quality in short-term perspective. A respondent from the IVO stated *“The sanctions and their financial consequences have forced the officials to compromise their important principles. For example in 2012, we have experienced a significant shortage in meats supply. We imported large quantities of meats from other countries without checking whether the animals were slaughtered according to religious and health standards. In addition, heavy weight chicken (7 kg) have been imported without assuring some essential health related criteria” (P15).*

Further problems as the non-standardized agriculturing and the shortages of food-producing animals were also linked to the sanctions. A manager at the IVO said *“Increasing costs reduces animal’s production and paves the way for the possible abuse. For example, one-day-old chicks were out of stock, raised and sold by a group of foreigners in illegal places which were totally out of our control system. It was unknown which drugs were fed to them, and we didn’t know whether withdrawal time was considered. Actually, it was unclear!” (P17).*

#### Sanctions and financing

##### Budget reduction for AMR policies

With the escalation of sanctions, the MOHME budget for AMR-related policies has been reduced. One of these policies was the establishment of the NCRUD. One member in the committee stated that *“We had sufficient budgets before these sanctions, later, most of these budgets, even for the clinically-severe cases were significantly cut. The budget of our committee is also still diminishing after sanctions as well” (P19).* In the same regard, one manager at the Iranian Society of Microbiology added *“The governmental revenues have reduced, which in turn resulted in the lower spending and less employment, meanwhile, we are already suffering from a shortage of manpower and work overload. For example, 10 technicians are currently working in our laboratory while we need 20 at least, and this may affect the quality of the results. At the end of the day, people themselves will be negatively influenced by these implications” (P23).*

##### More attention to the curative medicine

Participants from the both fields, human and veterinary medicine, believed that the scarcity of monetary resources due to the sanctions have drived the authorities to pay more attention to curative care, as it is more urgent, in budget allocation than prevention and infection control. One manager in the Deputy of Curative Affairs stated that *“Our hospitals have serious financial hardship, and these problems usually affect the infection prevention and control measures, e.g. it reduces the purchase of disinfectants. Even more recently, equipment are being re-used. The infection control and prevention should not be compromised in any condition” (P6).*

#### Sanctions and research and knowledge sharing

##### Quantity and quality of research on AMR

The lack of research budgets and the sanctions-induced inflation have squeezed AMR-related research. One researcher said *“Research needs money, facilities and communications, also a range of high quality and reliable materials, unfortunately, our work will be disrupted as they couldn’t be supplied. As a result, the outputs of our work will not be authentic nor feasible” (P21).* Another researcher added that *“We have to provide E-test for a research project but it was shocking for us because of its price. It became so expensive after the sanctions and then unaffordable” (P19).*

##### Researchers’ communication with global scientific community

Sanction-imposed reduction in the global diplomatic and economic collaborations with Iran has also adversely influenced the Iranian academic including AMR related- community. Sanctions have also disrupted the interactions of AMR researchers with their colleagues in other countries as well as with the international scientific organizations because of various difficulties such as visa, travel and residency expenses in those countries. In terms of publication, their work is experiencing various hurdles to be published in international journals. One of the managers at the Iranian Mycological Society said *“Unfortunately, we now have troubles getting our research published. They see the affiliation, immediate reject will be the result in most cases or at least with many restrictions. Many of my colleagues have lost contact with their international partners. Yet, they used both of their experience and equipment that was no longer possible” (P22).*

## Discussion

This study has sought to explain the consequences of international factors for the AMR management policies and practices in Iran. International factors were briefly found to be of both enabling and predisposing nature in relation to the AMR containment. The enabling factors included knowledge transfer and facilitation in the AMR policies agenda setting, formulation, implemention, and monitoring. Predisposing factors, instead, encompassed the migration of infectious patients, trafficking of medicine and livestock from the neighboring countries, and the imposed sanctions.

A review on the global health policies shows that the national and international political support for global health programs has not been equal, some receiving more support e.g. family planning and HIV/AIDS, some other less, e.g. malnutrition and pneumonia, despite of its high burden and severity [22].

The role of political and international factors in health has been always influential [14, 15, 23–25]. For instance, the role of diplomacy track in relation to the modern vaccine record seems impressive, e.g. in the eradication of smallpox and polio to some extent. A number of health related achievements emerged when the USA and USSR put their conflict aside during the Cold War. Similarly, the dialogue that took place between USA and Iran under the joint comprehensive plan of action (JCPOA) agreement between 2014 and 2016, opened the door for some accomplishments in public health [24]. However, following the exit of the USA from the JCPOA and initiation of new sanctions in 2018, such efforts were undermined [26].

AMR has topped the global agenda for the recent few years and is one highly affected by international interactions. The efforts of various actors ranging from NGOs, governmental bodies, and international organizations have made the issue well-known to the nonhealth and nonscientific communities and a special attention had been also paid to this issue from the international political bodies [12, 14]. Foreign actors similarly have played an active role in AMR policymaking in Iran along with, sustainable political support, effective policies, technology and efficient operating systems are essential to tackle this issue [22].

The imposed sanctions during the last 40 years have played a key role in relation to all health related activities [16–18, 20, 27], especially to the AMR efforts in the country; unilateral sanctions by the U.S.A. They have a destructive role in inability to supply the required vital elements for AMR contatinment. These sanctions are not a new issue for Iran as the internationally multilateral long-lasting sanctions imposed by the UN and the EU have begun in concurrent with the Iranian Revolution in 1979 [16, 18, 26]. Although the article 41 of the UN Charter authorizes the total or partial termination of economic sanctions with the targeted countries for the nonviolent measures, the adverse consequences of sanctions still persist especially in the case of AMR in Iran, as in the case of some other countries [13].

Although the countries imposing sanctions claim an exemption for health related products, the history of sanctions in different countries confirms that health systems are still adversely affected [17]. Sanctions may not directly target health systems, but the economic associated hardship has been affecting the Iranian health system to combat AMR. This is for instance, as to the poor capablility to access the sophisticated antimicrobial drugs produced through advanced technologies. In Iran, the 4% of imported drugs represent 40% of the total value of the Iranian pharmaceutical market [16, 27]. And also due to the sanctions and lack of medical products, diffusion disk is the widely-used test in Iran, and the MIC or E-test tests are performed only in few hospitals and research centers [28].

It should be noted that AMR is not just a problem that remains within the confines of medical centers. Arrival of just one patient with a resistant microorganism to a hospital may frustrate many years of protection and infection control strategies against AMR [12]. AMR is formed and developed in a complex cycle in different ecosystems, therefore, the global community should focus on its containment as a part of a broader agenda for universal health coverage and global health security [29]. Regular inter-sectoral and intra-sectoral meetings as an important mechanism for sharing information and experiences have been recommended by experts to improve the expertise of countries to bring the private sector and the scientific community together in order to overcome the AMR challenges by implementing the one-health approach [29].

Health diplomacy not only plays a key role in bringing this issue to the UN agenda, but also can help Iran to address the challenges posed by neighboring countries and sanctions. Though, it may might be hindered sometimes by sanctions. Iran should make use of health diplomacy to increase its power and exclude the trans-border challenges threatening the global security. The increased AMR among refugees, asylum seekers, refugee camps and detention facilities points out the need for improved life conditions, access to health care, and initiatives to facilitate detection of and appropriate high-quality treatment for AMR during transit and in host countries as well [30]. Along with strengthening infrastructure and financing such programs, keeping the door open to health diplomacy and international collaboration is essential for countries. The WHO country offices could contribute significantly in using the power of diplomacy [14].

Sanctions are also planned to isolate countries like Iran, and this sounds incompatible with the spirit of the GAP, as key informants also pointed to the sanctions’s effect on the Iran’s international research collaborations. This view is consistent with the literature which ensures inability of Iranian scholars to publish scientifc outputs, attend scientifc meetings, access to essential medical and laboratory supplies and information resources. They concluded that the academic boycotts violate researchers’ autonomy and curtail progress. Free exchange of ideas irrespective of creed is needed to optimize global scientifc progress [25].

Iranian policymakers need to think of ways to treat the vulnerability in order to confront these sanctions. For example, despite the embargo, Cuba has produced better health outcomes than most Latin American countries, and they are comparable to most of developed countries [23]. Emphasis on the primary care medicine, community health literacy, universal coverage, and accessibility to health services may explain the Cuban achievements in health sector and the attained health outcomes [23].

Evidenced-based decision making should be in place to ensure adopting best policies for supply, distribution, and administration of medicines, equipment and infrastructure based on the requirements and needs. Turning the threat into opportunity in the presence of drug shortage may open windows of opportunity to turn irrational use into a responsible use.

Our research was one of the first on AMR-related policies in Iran. However, our findings need to be interpreted with caution, as a long-term analysis is necessary to investigate the effects of international factors like sanctions on AMR in Iran. The perceived exaggerated emphasis on the sanction in the study is based on the participants’ perceptions. That is, the increasing intensity of international sanctions against Iran may have drawn the participants’ attentions more towards the sanctions and their implications. Surely, other national efforts, as rational use, are also key to handling AMR, which were outside of this study scope. Two related experts, due to their time constraints refused to participate.

## Conclusion

AMR is now turning into a global challenge and more or less all countries are facing with in some ways. LMICs including Iran are even more susceptible to be affected by AMR for many reasons. Therefore, international, regional and national collaborations are inevitably demanded to deal successfully with such an issue, given its nature. This study has sought to elaborate on the role of international factors in the AMR control and containment policies in Iran. As a result of the geopolitical situation of the country, a number of international factors, mainly sanctions, are affecting its policies including those related to the health sector and especially to AMR management. Although the sanctions are not allegedly targeting just the health system, the violation of people’s right to health is still their direct or indirect effect. In addition to the internal capability and efforts of the country, the international factors were also highly enabling and impowering in the AMR policy making and knowledge sharing, which should be seriously attended and reinforced. That is, tracing and reliance only on the internal capacities might not guarantee the success of AMR initiatives in Iran as the AMR control needs multi-sides cooperation. Health diplomacy might be an effective channel to ease the collaboration with the neighbours and non-neighbours. Disociation of health from politics and valuing humanitaring aspects of health could be of high use in the current management of AMR. Considering the concern of externality in health care, failure of any country to combat AMR will result in generating serious threats to global health, which should be alarming for international health stewarding organizations. Furthermore, future researches are recommended to identify potential strategies to improve the enabling factors and ways to alleviate sanctions effect on AMR control in countries.

## Supplementary information

**Additional file 1.**

**Additional file 2.**

## Data Availability

The datasets generated and analysed during the present study are available from the corresponding author on reasonable request.

## Abbreviations

AMR: Antimicrobial Resistance
WHO: World Health Organization
GAP: Global action plan
LMICS: Low and middle-income countries
NCRUD: National Committee of Rational Use of Drugs
ISIDTM: Iranian society of infection disease and tropical medicine
OIE: World Organisation for Animal Health
FAO: The Food and Agriculture Organization
MOHME: Ministry of Health and Medical Education
CAC: Codex Alimentarius Committee
GLASS: Global Antimicrobial Resistance Surveillance System
HRL: Health Reference Laboratory
CDC: Center for Communicable Disease Control
JCPOA: Joint Comprehensive Plan of Action
