# Supplementary material for: Control of antimicrobial resistance in Iran: the role of international factors
Source: BMC Public Health. 2020 Jun 5;20:873. doi: 10.1186/s12889-020-09006-8 (PMC7275379; doi:10.1186/s12889-020-09006-8)
Supplement: Supplementary file 1 — Additional file 1. [file 12889_2020_9006_MOESM1_ESM.docx]

M / F

- To start with, could you just tell me a little bit about who you are? )Profession / organization(
- Your name and affiliation are completely confidential and will not be published anywhere. You can also not answer a question at your discretion, or interrupt the interview.

1. What do you think are the important international and external determinants that have influenced Antimicrobial resistance in our country?

2. How did these factors affect (decrease or increase) AMR?

3. Have there been any of that have influenced the policies developed to combat AMR?

4. Has it been positive or helpful or has it had a detrimental effect on the policies that have been formed? How?

5. Which international actors and stakeholders have had a positive or negative impact on the AMR in our country? How?

6. is there anything else at all that you would like to add?

7. Would it be OK to contact you again?
