# Supplementary material for: Control of antimicrobial resistance in Iran: the role of international factors
Source: BMC Public Health. 2020 Jun 5;20:873. doi: 10.1186/s12889-020-09006-8 (PMC7275379; doi:10.1186/s12889-020-09006-8)
Supplement: Supplementary file 2 — Additional file 2. [file 12889_2020_9006_MOESM2_ESM.docx]

مرد / زن

- در ابتدا خودتان رو بیشتر معرفی بفرمایید؟ (شغل / سازمان مربوطه)
- نام و مشخصات و وابستگی سازمانی شما کاملا محرمانه است و هیچ جا منتشر نخواهد شد. همچنین شما می توانید در صورت صلاحدید به سوالی پاسخ ندهید، یا مصاحبه را قطع کنید.

1. به نظر شما مهمترین عوامل بین الملل و خارجی که بر مقاومت میکروبی در کشور ما تاثیر گذار بوده اند چه هستند؟
2. این عوامل بین المللی چگونه بر روی مقاومت میکروبی تاثیرگذار بوده اند (کاهش یا افزایش)؟
3. آیا مواردی بوده است که بر روی سیاستهای شکل گرفته برای مقابله با مقاومت میکروبی تاثیر گذار بوده باشند؟
4. این تاثیرات مثبت و کمک کننده بوده است یا بر روی سیاستهای شکل گرفته تاثیر مخرب داشته است؟ چگونه؟
5. کدامیک از بازیگران و ذینفعان بین المللی به مسئله مقاومت میکروبی در کشور ما تاثیر مثبت یا منفی داشته اند؟ چگونه؟
6. آیا نکته دیگه ای هم هست که بخواهید به مصاحبه اضافه بکنید؟
7. امکان پذیر است در صورت نیاز، مجددا با شما تماس بگیریم؟
